# In‐patient suicide after telephone delivery of bad news to a suspected COVID‐19 patient: What could be done to improve communication quality?

**DOI:** 10.1002/hcs2.74

**Published:** 2023-10-26

**Authors:** Natalie Tin Yau So, Olivia Miu Yung Ngan

**Affiliations:** ^1^ LKS Faculty of Medicine The University of Hong Kong Hong Kong SAR China; ^2^ Medical Ethics and Humanities Unit, School of Clinical Medicine The University of Hong Kong Hong Kong SAR China; ^3^ Centre for Medical Ethics and Law The University of Hong Kong Hong Kong SAR China

**Keywords:** cancer, communication, COVID‐19, patient suicide, ethics

**Abbreviations**
COVID-19: coronavirus disease 2019
CT Scan: computerised tomograph scan
PPE: personal protective equipment

## 1 INTRODUCTION

Breaking bad news is a critical communication competency for healthcare professionals. Any disclosure of a life‐threatening event, such as a malignancy diagnosis, often causes significant stress to patients. While some patients may respond with acceptance and a determination to fight their illness, research has consistently shown that cancer patients often respond to the disclosure of their diagnosis with a range of negative emotions, such as anxiety, distress, and depression [1, 2]. These reactions are often accompanied by feelings of fear, uncertainty, and a sense of loss of control over their lives. Patients may also experience denial, manifesting as reluctance to accept or discuss the diagnosis [3]. Avoidance is another common reaction, where patients may choose to avoid certain situations or people that remind them of their illness [4]. These reactions are not uncommon and are a natural response to the stress and uncertainty of cancer diagnosis.

A common ethical dilemma in breaking a cancer diagnosis is that patients have different preferences and coping mechanisms when dealing with difficult news, and it is important to explore their wish to know about their health condition. Some patients may want to be fully informed about their diagnosis, prognosis, and treatment options, as they believe it empowers them to make decisions and take control of their healthcare. They may also value the opportunity to prepare emotionally and practically for the challenges that lie ahead. However, other patients may prefer to shield themselves from the potentially distressing information [5]. They may prioritize maintaining hope, protecting their mental well‐being, or focusing on the present moment rather than dwelling on the future. Previous students showed that different cultures or religions influence how patients perceive the disease, their desire to know about the health condition, or their willingness to accept a diagnosis. For example, in some cultures, cancer is seen as a death sentence, leading to denial or avoidance of diagnosis and treatment [6]. There is a social stigma and gender label attached to cancer, which can lead to shame and embarrassment about the diagnosis [7, 8, 9]. Patients may be reluctant to seek medical attention, disclose their diagnosis, or follow through with treatment due to fear of being ostracized or discriminated against.

Remote communication methods like video and phone calls are being used more frequently to prevent the spread of the virus during disease outbreaks, such as the COVID‐19 pandemic. It has become more difficult for healthcare professionals to inform patients about their cancer diagnosis. However, giving a cancer diagnosis over the phone can be a challenge since it does not allow for in‐person support, and can come across as impersonal and insensitive. Unfortunately, in some cases, delivering bad news can have tragic consequences. One such example occurred during the COVID‐19 pandemic in Hong Kong, where an elderly patient, who had been hospitalized in an isolation ward, was informed of his malignancy diagnosis over the phone and subsequently suffocated to death using a plastic bag. This article will examine a real‐life suicide case in a hospital after a patient was informed of their cancer diagnosis via telephone and discuss the implications of telecommunication on breaking bad news [10].

## 2 CASE DISCUSSION OF A SUICIDAL CASE AFTER BREAKING THE CANCER DIAGNOSIS IN THE ISOLATION WARD

An elderly male (Mr. A) was admitted for shortness of breath, chest discomfort, and bilateral lower limb edema in 2022. A CT scan was performed, after which the patient and his wife were notified of a suspected diagnosis of metastatic lung cancer. He later became a close contact with COVID‐19 during his hospital stay and was transferred to an isolation cubicle. A disposable urinal in a plastic bag was provided. Blood tests for tumour markers later confirmed his cancer diagnosis and the on‐call doctor delivered the bad news to the patient by ward telephone shortly. Two days later, the patient was found unconscious in bed with his head covered in a plastic bag. Despite resuscitation, the patient eventually succumbed. During bereavement interviews, the patient's family recalled that the patient had expressed having pain and trouble sleeping.

The case covers several topics, including healthcare, patient care, cancer diagnosis, COVID‐19, isolation protocols, communication with patients and their families, and the importance of addressing pain and sleep issues in healthcare settings. There is no way to know, retrospectively, whether the doctor's choice to disclose the cancer diagnosis via ward telephone contributed to the patient's suicide. Nevertheless, it is worth discussing the appropriacy of breaking bad news via telephone in a hospital setting. This tragic incident highlights the importance of proper patient care and communication during hospital stays.

### 2.1 Cancer disclosure: Telecommunication versus in‐person disclosure

Patients in a typical clinical environment rely on their doctors to provide personalized information, explain treatment options, and help them comprehend their circumstances to make informed decisions. Doctors are expected to deliver unpleasant news, but their role goes beyond merely revealing diagnoses in clinical settings. An ethical predicament persists until patients indicate their preference on how much information they want to receive about their medical condition. Determining whether patients want to be fully or partially informed or kept in the dark is crucial. This approach ensures doctors adhere to ethical principles such as respecting patients' autonomy and promoting beneficence. This is especially important in view of a recent meta‐analysis which discovered that in Chinese culture, doctors and caregivers often refrain from disclosing severe medical conditions, such as cancer, to patients to protect their psychological well‐being [11]. Even if doctors choose not to disclose a diagnosis or prognosis, they should uphold ethical standards by following patients' wishes. They must possess the necessary skills to deliver bad news in a sensitive manner, particularly in cultures where nondisclosure is prevalent.

During the pandemic, many nonemergent clinical services were suspended, and infection control considerations contributed to a sharp rise in the use of telemedicine to deliver in‐patient healthcare services [12]. This raises questions about the effectiveness of breaking bad news remotely, as opposed to the traditional face‐to‐face model. Breaking bad news requires immense skill and care because patients often experience symptoms of anxiety and depression after receiving a cancer diagnosis [13] and different models for breaking bad news have been implemented to minimize psychological harm to patients. This includes the widely‐used SPIKES protocol, which emphasizes the need for doctors to address patients' emotions with empathetic responses [14]. Before the pandemic, the protocol emphasized that bad news should not be delivered over the phone [15], since telemedicine adds complexity to communication in healthcare. It is challenging to assess patients' emotional states and deliver appropriate responses remotely, and it has been found that physicians lack the skills to break bad news, especially when using nonphysical ways during the pandemic [16]. Human communication depends heavily on body language and paralinguistic cues [17], and restrictions of physical contact in telemedicine make it difficult for healthcare professionals to act appropriately in response to patients' emotions. In addition, patients also prefer receiving bad news in person without physical barriers [18]. It is, therefore, essential for clinicians to carefully consider how the use of telemedicine might affect the quality of breaking bad news, even during times when in‐person communication may be difficult.

The importance of considering patients' emotional states was especially relevant during the COVID‐19 pandemic when mental health declined across different populations. During the pandemic, the prevalence of depressive symptoms in Hong Kong doubled compared to 2016 and 2017, while anxiety increased by 42.3%. A significant increase in stress levels was noticed, particularly among older adults [19]. The exacerbation in mental health conditions may be explained by lockdowns and home confinement during the pandemic [20], and such mitigation policies have also been found to contribute to factors known to precipitate suicide, such as social isolation, loneliness, and financial stressors [21, 22]. The pandemic was a period during which patients may be particularly susceptible to emotional stress, further highlighting the need for doctors to break bad news skillfully and empathetically.

Mr. A's hospital admission occurred during the second quarter of 2022 when there were effective clinical protocols for in‐hospital use of personal protective equipment (PPE) to protect against the SARS‐CoV‐2 virus [23]. Provided that the appropriate PPE were available, it would have been desirable for the bad news to be delivered in person. The fact that the patient was physically located within the hospital supports in‐person delivery of the diagnosis. However, if PPE were scarce, the need to conserve PPE would have to be considered against the benefit of breaking bad news face‐to‐face. While this is a decision to be made by the doctor, efforts to conserve resources must not be at the expense of a significant compromise to patient care. This is especially true if the doctor could have anticipated an inferior telephone consultation quality. Although resource allocation is inevitable and doctors often feel the need to make choices based on resource availability, doctors should act primarily in the interests of their patients because the individual doctor‐patient relationship is the bedrock of bedside clinical ethics [24]. Decisions about resource allocation should more appropriately be left to institutions, which, through developing guidelines and protocols, are better positioned than individual doctors in allocating scarce resources. How the diagnosis was delivered to Mr. A through a ward telephone may or may not have contributed to his suicide, and we cannot speculate on this. This case underscores the need for healthcare professionals to possess the necessary skills to deliver bad news sensitively and compassionately, particularly in cultures where nondisclosure is prevalent. Additionally, it highlights the importance of addressing patients' psychological and emotional needs when disclosing a cancer diagnosis.

### 2.2 Delaying disclosure of a cancer diagnosis

Infection control measures during the COVID‐19 pandemic indeed impeded doctors' ability to meet with patients in person [25]. For discussion's sake, let us assume that the doctor could not have met with Mr. A in person while he was in isolation. The doctor would either have to break the bad news remotely or wait until Mr. A was out of isolation to discuss it in person. The harms of delaying disclosure of the cancer diagnosis must, therefore, be balanced against the detriments of breaking bad news remotely—how long should the doctor wait until the need to disclose the information outweighs the disadvantage of breaking bad news remotely? Two main factors should be considered. First, if a delay in disclosure affects the patient's cancer management, the patient should be notified of their cancer diagnosis as soon as possible for further investigations and treatment. Second, if the patient is already aware of a suspected malignancy, there is a stronger incentive for prompt disclosure of the cancer diagnosis. It has been found that the prevalence of anxiety is high among patients awaiting diagnostic procedures for cancer. The time when patients are waiting for a cancer diagnosis is often associated with increased anxiety symptoms [2]. Furthermore, patients expect test results to be returned within a short period and may feel “ignored, resentful, and sometimes frightened” if they do not receive their results promptly [26]. There are substantial grounds to suggest that once a cancer diagnosis is made, it should be conveyed to the patient as soon as possible to minimize the negative emotions patients experience while waiting.

In Mr. A's case, it is unlikely that delaying the disclosure of his diagnosis until the end of his isolation period would have affected his cancer management. He would likely have had to complete his isolation before further investigations or treatment could be given, and this may mean that there was no pressing need to break bad news. However, before being isolated, Mr. A was told that his CT findings were suggestive of metastatic lung cancer, and he was waiting for laboratory investigations to confirm the diagnosis. A few hours can seem like an eternity to patients awaiting confirmatory results. Since the doctor had already made the diagnosis, it is questionable whether the diagnosis should be withheld from Mr. A until he was no longer under isolation. This scenario differs from one in which an unsuspecting patient is told, out of the blue, that they have metastatic cancer. Patients are found to have fewer anxiety symptoms after receiving a cancer diagnosis if they had been prepared for a possible cancer diagnosis. Since Mr. A had already been told about a suspected malignancy, this may support the doctor's decision to promptly tell Mr. A about his diagnosis, even if this had to be done over the telephone. Balancing the harms of delaying disclosure against the risks of breaking bad news remotely is no easy task—it requires a thorough understanding of the patient's clinical condition, prognosis, expected management plan, and the patient's information preferences and emotional state. As such, clinicians should seek a holistic understanding of patients' conditions before breaking bad news to minimize the harm of delaying disclosure or breaking bad news remotely.

The case also raises concerns regarding the role of the patient's family in the decision‐making process. It is not mentioned whether the doctors approached Mr. A's family to discuss his medical condition and treatment options. In Chinese culture, family members often play a vital role in decision‐making regarding medical treatment and end‐of‐life care, and it is also common for doctors and caregivers to avoid revealing severe medical conditions to protect patients' psychological well‐being. Therefore, it is crucial for healthcare professionals to engage with patients' families and involve them in the decision‐making process, particularly when discussing a cancer diagnosis and the treatment options available. By involving the family in the decision‐making process, healthcare professionals may better ensure that the patient's wishes and values are respected and that the patient receives the necessary emotional and psychological support. This approach aligns with the ethical principles of beneficence, nonmaleficence, and respect for patient autonomy. Healthcare professionals need to understand and respect the cultural values and beliefs of their patients and their families to provide patient‐centered care.

## 3 POSSIBLE WAYS FORWARD

The COVID‐19 pandemic has demonstrated that telecommunication may be necessary when infection control concerns render traditional in‐person consultations less preferable. How, then, might we adapt such that bad news can be broken remotely empathetically and skilfully? Various authors have contributed to the discussion on adjustments that can be made to improve remote communication of bad news during the COVID‐19 era. Landa‐Ramirez et al. proposed a systematic tool to help healthcare providers deliver bad news virtually [27], while Vitto et al. and Gonçalves Júnior, Jucier et al. offered ways in which the SPIKES protocol can be modified to better meet patients' needs during virtual delivery of bad news [15, 28]. Mr. A's cancer diagnosis was delivered via ward telephone. If possible, a communication device with video and audio, such as a smartphone or tablet, is preferred over audio‐only communication [29].

Research conducted across different clinical settings has demonstrated that telecommunication with both video and audio is considered superior to audio‐only teleconsultations in building rapport, providing visual cues and reassurance, and enhancing communication [30, 31, 32]. This sentiment is also shared by clinicians and patients' family members, who believe phone calls are helpful for brief updates. In contrast, video calls are preferable for aligning clinician and family perspectives [24]. Furthermore, it is essential to consider many nonclinical factors that may affect information delivery, such as patient health literacy, religion, social‐cultural practice, and language barriers. Relational autonomy is a dominant culture and value in Hong Kong, and family involvement in consultations is highly valued [33]. Allowing the patient to include their loved ones in remote conversations is crucial, especially if the patient is undergoing isolation and has limited opportunities to connect with others. Given the emotionally challenging nature of receiving a cancer diagnosis virtually while being in isolation, mental health professionals or palliative care specialists should also be involved, if necessary, to assist the patient in navigating through the process [1].

## 4 CONCLUSION

Bad news should be delivered in person whenever possible in a clinical setting. When circumstances prohibit information from being delivered promptly and face‐to‐face, the benefits of breaking bad news in person must be balanced against the disadvantages of delaying information disclosure, which requires a holistic understanding of patients' needs. The tragic incident prompted reflection on the proper use of digitalized technology in the burgeoning telehealth system as a means of health communication. Telemedicine complicates communication in healthcare settings, especially when breaking bad news. Before incorporating teleconsultation as a regular clinical service, individual characteristics (e.g., empathetic listening and observation skills among healthcare providers) and organizational readiness (operational barriers, patient safety, and privacy settings) to adopt videoconferencing should be reviewed and assessed in the local context. This is important in view of global trends to increase the use of telecommunication in healthcare settings and anticipation of future events, such as pandemics, which may necessitate widespread application of telehealth.

## AUTHOR CONTRIBUTIONS

The authors contributed equally to the drafting and revision of the manuscript.

## CONFLICT OF INTEREST

The authors declare no conflict of interest.

## ETHICS STATEMENT

This study is a theoretical discussion and does not require ethics clearance.

## INFORMED CONSENT

This study does not involve human research participants, and therefore no informed consent was obtained.

## Data Availability

Data sharing is not applicable to this article as no data sets were generated or analyzed during the current study.
